# Critical and steady-state characteristics of delay propagation in an airport network

**DOI:** 10.1371/journal.pone.0288200

**Published:** 2023-07-07

**Authors:** Hong-Guang Yao, Hang Zhang

**Affiliations:** College of Aviation Transportation, Shanghai University of Engineering Science, Shanghai, China; National University of Sciences and Technology NUST, PAKISTAN

## Abstract

In this work, we established a density equation for delayed airports to investigate the horizontal propagation mechanism of delays among airports in an airport network. We explored the critical conditions, steady-state features, and scale of the delay propagation, and designed a simulation system to verify the accuracy of the results. The results indicated that, due to the no-table scale-free feature of an airport network, the critical value of delay propagation is extremely small, and delays are prone to propagate among airports. Furthermore, as delay propagation reaches a steady state in an aviation network, the degree value of the node becomes highly correlated with its delay state. Hub airports with high degree values are the most prone to being affected by delay propagation. In addition, the number of airports that are initially delayed influences the time required for delay propagation to reach a steady state. Specifically, if there are fewer initially delayed airports, a longer time is required to reach a steady state. In the steady state, the delay ratios of airports with different degree values in the network converge to a balance point. The delay degree of the node is highly positively correlated with the delay propagation rate in the network, but negatively related to the degree distribution index of the network.

## Introduction

Flight delays have long been known to lead to lower passenger satisfaction and lower airline operational efficiency. A single airplane must perform multiple flight tasks every day to maintain airline efficiency and reduce operating costs. The flight chain of an airplane is fixed in advance. Consequently, the delay of any one flight will affect later flights, and this triggers a chain reaction, leading to further delays in the flight schedule. Flights that are executed later are often delayed for a longer period of time [1]. This is known as the propagation of a vertical flight delay within an airline. Moreover, numerous flights converge at the airport, and they await takeoff and landing according to a specified order. Due to the exclusivity of stands and runways in use, the arrival and departure of a delayed flight will disrupt the schedule of the airport and bring about an imbalance between the supply and demand of multiple flight resources, including airport capacity and air flow [2]. Consequently, other flights are then influenced and delayed. This is known as horizontal flight delay propagation among airports.

Air transport is currently operated through a network. The airport network is complex and consists of mutually interacting and correlated aviation transport resources including airports, routes, transport capacity, and airspace resources [3]. Airports and the routes connecting them form the basic structure of the airport network. The serviceability of the network depends on transport availability and airspace capacity [4]. After a flight delay begins to propagate horizontally in airports, the structural features of the airport network and flight operation rules act in concert and render the propagation complicated. However, the in-flight delay can be partially absorbed or even eliminated. For example, the delay can be partially mitigated by good flying conditions because the flight time shown in the flight schedule is often greater than the minimum flight time required. In addition, if the turnaround time of later flights can be minimized so as to be shorter than the planned dwell time, then the time difference can alleviate the time delay. In the case of a short initial delay time, the delay propagation is eliminated in the airport network after several absorptions [5]. However, an airport that reaches its capacity limit has poor performance in terms of absorbing the delay time and scheduling aviation resources. After a flight delay occurs, the coupling effect of the network nodes leads to a spatial and temporal imbalance between the supply and demand of aviation transportation resources. The delay propagates to downstream flights and airports, and initiates a chain reaction. Consequently, the number of delayed flights and the delay time are multiplied, and in serious cases, the entire airport network may even be paralyzed. In particular, increasingly dense flights and nearly saturated airspace flows worsen the problem of flight delay propagation [6]. Therefore, the solution to flight delay is to prevent and address flight delay propagation among airports.

Early research on flight delay propagation focused on its “causes, power sources, and chain propagation channels.” As discussed above, propagation is a special property of flight delays [7]. Only 30% of the total flight delays in China are caused by a force majeure, such as weather, whereas over 60% of flight delays result from delay propagation [8]. Flight delay propagation significantly affects the social economy. It has been found that flight delays indirectly caused economic losses of 350.71 billion yuan in China in 2013 alone. This demonstrates the importance of mitigating flight delays [9]. Flight operation is the result of the coordination of diversified flight resources, and as such, it is therefore influenced by flight resources. When a delay occurs, it disrupts the entire system, resulting in delay propagation [10]. Technical methods, such as queuing theory, can be used to analyze the delay status of downstream flights in the flight chain after flight delays occur. These methods can elucidate the “chain reaction” of delay propagation [11]. To ensure an on-time flight, it is necessary for airlines, airports, air control, and other parties to cooperate, and over 20 types of sufficient flight resources need to be supplied in time [12]. Flight delay propagation is affected and limited by numerous microcosmic factors, and its mechanism is complex. However, the airport network structure is an essential factor that affects flight delay propagation [13]. Thus, investigation of flight delay propagation mechanisms can facilitate the development of effective approaches to prevent flight delay propagation and reduce flight delay loss. This research can aid the air transport management department in providing theoretical guidance for flight delay treatment [14].

As the causes and modes of flight delay propagation have been clarified, researchers have begun to focus on minimizing the influence of flight delay propagation and rapidly restoring the flight operation order. The premise of delay propagation treatment is to accurately predict the effect of flight delay propagation on subsequent flights. A delay evaluation tool was proposed to assess the delayed accumulation of flights downstream of an initially delayed flight caused by crew or aircraft factors [15]. To accurately reflect the process of delay propagation in successive flights, simulation experiments with a large amount of data were carried out to examine the propagation pattern of the delay time in successive flights [16]. A Bayesian network [17], Petri net [18], and other mathematic analysis models [19, 20] were used to simulate and analyze flight delay propagation waves and effects, explore the distribution and flow of multiple resources required during flight, and evaluate the mutual influence between flights, airports, delay chain waves, and the propagation process. A chain reaction model of flight delays was designed to determine the optimal allocation scheme of the flight resources for different targets after the flight delay propagates. The results indicated that the sensitivity of the predetermined route to the variable flight interval in the terminal area can be eliminated by reducing the initial delay [21], dynamically changing the flight vortex separation standard of the runway [22], and improving the operational efficiency of the airport terminal [23].

The previous studies summarized above highlighted the flight chain delay propagation issue within one airline or airport, and were aimed at investigating the influence of the flight delay on downstream flights [24, 25], solving the problem of the delay robustness of flight schedule preparation [26, 27], optimizing the allocation of flight resources of airlines or airports [28, 29], and rapidly restoring delayed flights [30]. However, in reality, horizontal propagation of flight delays among airports occurs more frequently than vertical propagation within one airport. The delay spreads to flights of downstream airports through the coupling effect between network nodes. Thus, flight delay propagation generally involves more than one airline. To prevent flight delay propagation, it is therefore necessary to analyze the structural features of airport networks in depth, and the complex relationship between delayed flights and various transportation resources should be comprehensively studied.

Complex network transmission dynamics has emerged as a new technical means to study the transmission and propagation problem in a network and has been used successfully in the study of infectious disease transmission [31, 32], computer virus transmission [33], and delay and congestion propagation in transportation networks [34, 35]. The topological structure of a network remarkably affects transmission and propagation, and a small-world network allows for far more rapid and easier transmission and propagation compared to a regular network [36]. A scale-free network permits a greater transmission intensity. Furthermore, even if the transmission intensity is low, the transmission can be sustained in the network [37]. In addition, both the transmission and propagation in the network have a critical value for the load, which is associated with the topological structure of the network. When the load of nodes or edges in the network surpasses a critical value, a significant amount of flight delay propagation occurs [38, 39]. Further studies have shown that there is a critical value of nodes in a network for the delay propagation time [40, 41]. Complex network theory provides a new modeling approach to examining delay propagation in urban transportation, aviation, and logistics networks. According to complex network theory, the rules are first established, based on which the system evolves spontaneously. The impact of the network structure on system evolution is then analyzed. This illustrates how to model complex systems [42]. A model built based on the complex network theory enables qualitative and quantitative analysis of the delay dynamic propagation in the network and allows for investigation of the propagation pattern. Hence, the model is critical to effectively mitigating delay propagation [43].

Unlike conducting research on flight delay diffusion from the perspective of a single airline or airport, we take the airport network as the object, simulate the delay diffusion process by establishing a SIS propagation model with flights as the medium, determine critical conditions, and analyze steady-state characteristics. Unlike traditional SIS models that overlook the characteristics of propagation media, the flight delay diffusion model established in this article fully considers the role of flights as propagation media in delay diffusion, and takes the number of delayed flights as an important factor in the probability of delay occurring at the destination airport. In addition, considering the significant scale-free characteristics of the airport network, the distribution of flights in the network is also closely related to the network structure. In general, flight distribution is an important factor affecting the spread of downstream airport delays. Airports with high degree values often have more flight volumes. Therefore, in the model established in this article, the number of flights is set based on the degree value of the airport, making the model closer to the real situation.

This article focuses on the characteristics of flight delay propagation in airport networks from the beginning to the stable state. Due to the scale-free characteristics of airport networks, homogeneous SIS models cannot accurately reflect flight delay propagation. Therefore, this article groups airports according to degree values and studies the relationship between the density of delayed airports and degree values and delay propagation time. Analyzed the range of values for delayed airport density and the convergence interval of airport delay proportions with different degree values. The scale of airports with flight delays in the airport network was studied. The accuracy of the research results was verified through the flight delay propagation data and simulation system of the Chinese airport network; Finally, the main conclusions were summarized.

## Theory and method

### Analysis of the mean-field theory

Airports are the nodes and routes are the edges in an airport network structure. Thus, airports are not only the basic units of an airport network but also a medium for flight delay propagation. An increased number of delayed airports in a network is therefore a reflection of the delay propagation mechanism.

Airports have normal and delayed statuses. An airport is considered “normal” if all flights arrive and leave on schedule, whereas an airport is considered “delayed” if arrival and departure flights are suspended. In an airport network, a delayed airport may affect other airports through their respective routes. Under certain conditions, a normal airport may be delayed and a delayed airport may be restored to normal.

In an airport network, flight operation is achieved through the cooperation of multiple parties, including airlines, airports, and air control. Whether the delay of one flight affects other flights in the airport is influenced by micro factors, such as airport runways, airspaces, and ground service resources. In airport networks, the flight resources of airports differ significantly. In addition, there are a large number of diversified flight resources that ensure the normal operation of flights at different times, and they are mutually influenced. The delays propagation in the airport network is influenced by many uncertain factors. Whether a certain airport will be affected by delay propagation is uncertain and presents an unpredictable disorderly state. However, both the number of delayed airports and the delay propagation exhibit an evident regular change trend from the viewpoint of the entire network. In other words, delay propagation in an airport network is generally disordered locally and in an orderly manner.

The mean-field theory was first proposed to study molecular thermodynamics, and it serves as theoretical support for analyzing a complex system that is disordered locally and orderly in general [44]. The mean-field theory can elucidate the development of propagation dynamics in complex networks. The core idea of this theory is that the total effect of interactions between all the basic elements of all scales plays a leading role in a change from a relatively disordered state to a relatively orderly state. However, the “local information” in the surrounding environment of each basic element is trivial [45]. Thus, the micro factors for an airport can be ignored, and only the global and average flight delay propagation possibility across the entire airport network is considered. In other words, only the effective flight delay propagation rate *λ*, which is described as a constant parameter, is used to evaluate flight delay propagation among airports.

### Construction of SIS model for delay propagation

The change pattern in airport delay status in an airport network is “normal (*S*) → delayed (*I*) → normal (*S*).” According to the mean-field theory, delay propagation in an airport network as a whole is regarded as a mean field after ignoring the uncertainty of the airport status. The probability that an airport changes from “normal” to “delayed” states is set as *v*, and the probability that it is restored from “delayed” to “normal” states is set as *δ*. The delay propagation rate *λ* can then be represented by the ratio of *v* and *δ*, as shown in Formula (1):

$$\lambda =\frac{v}{\delta }$$ (1)

where *s(t)* and *i(t)* represent the density of the nodes in the airport network in the *S* and *I* states, respectively, at time *t*. the airport status in the network can then be expressed by the differential equations shown in Formula (2):

$$\{\begin{array}{c} \frac{ds(t)}{dt}=-v\times i(t)+\delta \times i(t)\\ \frac{di(t)}{dt}=v\times i(t)-\delta \times i(t) \end{array}$$ (2)

where the two equations represent the densities of normal and delayed airports at time *t* in the network. *v×i(t)* denotes the variation in the airport density changing from a normal state to a delayed state. The increased number of delayed airports is directly proportional to the number of delayed airports and the flight delay propagation rate at *t*. *δ×i(t)* indicates the variation in the density of airports changing from a delayed state to a normal state.

In these equations, the critical point *λ*_*c*_ exists between *v* and *δ*. When *v* < *λ*_*c*_*δ*, a greater number of airports in the network are restored from a delayed state compared to the number of airports changing to a delayed state, and the steady-state solution of the equations is *i(T)* = 0. When *v* > *λ*_*c*_*δ*, an increasing number of nodes is delayed, and the steady-state solution of the equations is *i(T) >* 0. When *v* = *λ*_*c*_*δ*, the increased number of delayed nodes is equal to that of normal nodes.

### Determination of the critical value

#### Structural feature analysis of an airport network

Numerous empirical studies in recent years have revealed the typical scale-free features of an airport network [46–48]. The effect and role of each airport in a network differ markedly, and the difference exists mainly in the route link to other airports. Specifically, a few key hub airports are connected to a substantial number of other airports by routes, whereas most of the airports are rarely linked to other airports by routes. Thus, the key hub airports play a dominant role in airport networks [49].

The degree value, *k*_*i*_, of the nodes in the network is defined as the total number of edges connected to node *i*. In the network, a node with a larger degree value is often connected to more nodes and has more edges. This node has notable effects on the network [50]. The average degree value *k*_*i*_ for node *i* in the network is the average degree of < *k* >.

Degree distribution is an essential indicator of the scale-free attribute of a network [51]. The degree distribution denotes the probability that the degree value of a random node equals *k*, and this probability can be represented by the distribution function *P(k)*. The degree distribution of a network can be expressed by the power-law distribution formula *P(k)∝k*^*-γ*^. To facilitate this research, a cumulative distribution function is frequently used to reflect the degree distribution. The cumulative distribution function shows the probability distribution of nodes that have degree values larger than *k*. If the degree distribution of the nodes in the network conforms to the power law distribution pattern, then *P(k)∝k*^*-γ*^. Otherwise, the network is scale free. *γ* is also known as the degree-distribution index. A smaller *γ* indicates a larger difference in the degree values of the nodes and a stronger scale-free attribute [52].

In a scale-free network, the degree distribution of the nodes follows a power-law distribution pattern. Therefore, nodes with larger degree values are connected to more airports by routes and are more easily influenced, compared with other nodes [53]. Consequently, airports that are part of an airport network with a large total degree value are more likely to be delayed once the flight delay propagates. Moreover, airports with a large degree of value have more routes, and once they are delayed, a greater number of flights will be affected. This aggravates the spatial and temporal imbalance of the flight resource distribution in the airport network and accelerates delay propagation. However, delay propagation can be absorbed and even eliminated by downstream flights, and it is not definite that the delay will increase the number of delayed airports. Hence, a critical condition exists for flight delays in airport networks. Only when the critical condition is reached or exceeded will the delay propagation affect the entire network. The study of the critical condition for flight delay propagation is of great importance to reveal the flight delay propagation mechanism in airport networks and reduce flight delay propagation loss.

#### Establishment of the delayed airport density equation

The delayed airport density equation can be used to calculate the critical value of the delay propagation. To facilitate this research, the delay propagation of airports in the airport network and its critical conditions were studied. Because the delay propagates through the operating flights in the airport network, the influence of the medium flow on delay propagation should be highlighted when establishing the delay propagation equation.

The proportion of delayed nodes to all nodes at *t* in an airport network is taken as the density of the delayed nodes *i(t)*. When *t* is sufficiently long, the number of delayed nodes is dynamically stable. The steady-state density is denoted by *i*. To accurately reflect the influence of the network topology on delay propagation, airports are divided into different groups according to their degree values. Airports with the same degree of value *k* are classified into the same group. *i*_*k*_*(t)* represents the density of delayed airports in the group with degree value *k*. The density equation of delayed airports with a degree value of *k* is obtained based on mean-field theory, as shown in Formula (3).

$$\frac{di_{k}(t)}{dt}=-i_{k}(t)\delta +vk\frac{wk}{\sum_{i=1}k_{i}}(1-i_{k}(t))\sum_{k'}P(k'|k)i_{k}(t)$$ (3)

where *k* is the number of nodes connected to the node, namely, the degree value, *W* is the average number of flights in an airport network, *N* is the number of airports in an airport network, *k*_*i*_ is the degree value of node *i* in the network, *v* is the probability that normal nodes are delayed due to the influence of the delayed nodes per unit time, and *δ* is the probability that the delayed nodes return to normal per unit time.

-*i*_*k*_*(t)δ* represents the density of airports restored from the delayed state to the normal state at time *t*.

$vk\frac{wk}{\sum_{i=1}k_{i}}(1-i_{k}(t))\sum_{k'}P(k'|k)i_{k}(t)$ is the density of airports that changes from a normal state to a delayed state at time *t*. It is the product of the infection rate *v*, the number of adjacent airports *k*, the number of flights at the airport $\frac{wk}{\sum_{i=1}k_{i}}$, and $\sum_{k'}P(k'|k)i_{k}(t)$. $\sum_{k'}P(k'|k)i_{k}(t)$ is the sum of the density of airports vulnerable to the delay impact and the joint probability of connection between nodes with degree values of *k* and *$k^{\prime }$*.

#### Critical condition solution of delay propagation

If <*k*> is used to describe the average degree of airports in the airport network, then $\frac{wk}{\sum_{i=1}k_{i}}\text{=}\frac{\mathrm{wk}}{N<k>}$. Let $\sum_{k'}P(k'|k)i_{k}(t)$ be Θ(*i*(*t*)), and substitute $\lambda =\frac{v}{\delta }$ into Formula (3). Then, we have

$$\frac{di_{k}(t)}{dt}=-i_{k}(t)+\lambda k\frac{wk}{N<k>}(1-i_{k}(t))\Theta (i(t))$$ (4)

When the duration of time *t* is long, the delayed airport density reaches a steady state in an airport network. Because *i* is a function of *λ*,Θ is also a function of *λ*. Deriving *t* on the left and right sides of Formula (4), and letting *∂_t_i_k_*(*t*) = 0, we have

$$i_{k}=\frac{Wk^{2}\lambda \Theta }{Wk^{2}\lambda \Theta +N<k>}$$ (5)

The average degree of nodes is denoted by <*k*> . Because P(k'|k)=kP(k)〈k〉, Θ can be written as

$$\Theta =\sum_{k'}P(k'|k)i_{k}=\frac{1}{\langle k\rangle }\sum_{k}kP(k)\frac{Wk^{2}\lambda \Theta }{Wk^{2}\lambda \Theta +N<k>}$$ (6)

Formula (6) has a trivial solution: Θ = 0. If delay propagation occurs in an airport network, the number of delayed nodes increases at *t*. Therefore, to make Formula (6) have a nontrivial solution, Θ > 0, Formula (6) must satisfy

$$\frac{<k>}{\lambda }=\sum_{k}\frac{P(k)Wk^{3}\Theta }{N<k>+Wk^{2}\lambda }$$ (7)

According to the Taylor expansion,

$$\frac{1}{1\text{+}Wk^{2}\lambda \Theta /N<k>}=\sum_{j=0}^{\infty }{\left(-1\right)}^{j}{\left(Wk^{2}\lambda \Theta /N<k>\right)}^{j}$$ (8)

Formula (7) can be expanded and derived as follows: For all *k* values, if *Wk*^2^*λ*Θ/*N* < *k* > < 1 (namely $\Theta <\frac{N<k>}{W\lambda k_{\max}^{2}}$), Formula (9) can be obtained:

$$\frac{<k>}{\lambda }=\sum_{j=0}^{\infty }{\left(-1\right)}^{j}\{\sum_{k}P(k)Wk^{j+3}/N<k>\}\lambda^{j}\Theta^{j}=\sum_{j=0}^{\infty }{\left(-1\right)}^{j}<k^{j+3}>\frac{W}{N<k>}\lambda^{j}\Theta^{j}$$ (9)

When $\Theta <\frac{N<k>}{W\lambda k_{\max}^{2}}$, Formula (9) converges in sequence. Because Θ = 0 when *λ* = 0, the nontrivial solution Θ > 0 exists only when *λ* > *λ*_*c*_ ≥ 0, according to the definition of the critical value of the flight delay propagation. When Θ > 0 and it is so small that it meets the condition $\Theta <\frac{N<k>}{W\lambda k_{max}^{2}}$, Formula (9) can be expanded, and its second order equation is obtained:

$$\frac{<k>}{\lambda }=<k^{3}>\frac{W}{N<k>}-<k^{4}>\frac{W}{N<k>}\lambda \Theta +<k^{5}>\frac{W}{N<k>}\lambda^{2}\Theta^{2}$$ (10)

When *λ > λ*_*c*_ and Θ are extremely small, Formula (10) can be simplified as

$$\frac{<k>}{\lambda }=<k^{3}>\frac{W}{N<k>}$$ (11)

The critical value of the flight delay propagation in an airport network can be calculated by

$$\lambda >\lambda_{c}=\frac{N<k{>}^{2}}{W<k^{3}>}$$ (12)

where <*k*^3^> is the average of the cubes of node degree values in the network, and <*k*>^2^ is the square of the average degree values of nodes.

It can be deduced from Formula (12) that, because an airport network is remarkably scale-free, *W<k*^*3*^*>* is far greater than *N<k>*^*2*^. This indicates that the critical value *λ*_c_ of the delay propagation is small, and delay propagation can easily occur in airport networks.

## Results

When the critical conditions are reached, delayed flights will trigger a reallocation of air transportation resources at the destination airport, thereby increasing the probability of flight delays at other airports, forming a flight delay propagation at a few airports throughout the entire network. After the flight delay propagation, overall, the number of delayed airports in the network will continue to increase. As the number of delayed airports increases, it will have a suppressive effect on flights executing flight tasks, thereby reducing the number of flights per unit time in the network, alleviating the service pressure on the airport network, and accelerating the speed at which the airport returns from a delayed state to a normal state. Therefore, when the flight delay propagation in the network reaches a certain level, the number of delayed airports in the network will maintain a relatively stable level, which is called the stable state of flight delay propagation in the airport network.

Studying the characteristics of the stable state of flight delay propagation in the airport network is the key to achieving flight delay propagation governance. Due to the scale-free characteristics of the airport network, a flight delay propagation model was established by grouping airports according to degree values. Therefore, the stable state characteristics of flight delay propagation in the network can be reflected from the perspective of degree values and delay airport density. We will focus on studying the relationship between airport degree values and delay density, the range of delay airport density values, the convergence interval of delay airport density with different degree values, and the factors affecting the scale of flight delay airports in the network.

### Analysis of delay propagation in the steady state

#### Analysis of Θ Properties in the steady state

According to Formula (6), Θ can be further simplified into

$$\Theta =\sum_{k}kP(k)<k{>}^{-1}\frac{Wk^{2}\lambda \Theta }{Wk^{2}\lambda \Theta +N<k>}$$ (13)

The non-trivial solution of (13) is equivalent to that of the following equation:

$$1=\sum_{k}kP(k)<k{>}^{-1}\frac{Wk^{2}\lambda }{Wk^{2}\lambda \Theta +N<k>},\Theta >0$$ (14)

Let $g(\Theta )=\sum_{k}kP(k)<k{>}^{-1}\frac{Wk^{2}\lambda }{Wk^{2}\lambda \Theta +N<k>}$, and it is evident that the continuous function *g*(Θ) strictly decreases at Θ ≥ 0. $g(1)=\sum_{k}kP(k)<k{>}^{-1}\frac{Wk^{2}\lambda }{Wk^{2}\lambda +N<k>}<\sum_{k}kP(k)<k{>}^{-1}=1$, and $g(0)=\sum_{k}kP(k)<k{>}^{-1}\frac{Wk^{2}\lambda }{N<k>}\text{=}\lambda \frac{W<k^{3}>}{N<k{>}^{2}}$. The necessary and sufficient condition for Formula (14) with a non-trivial solution Θ > 0 is $g(0)=\lambda \frac{W<k^{3}>}{N<k{>}^{2}}>1$, namely $\lambda >\frac{N<k{>}^{2}}{W<k^{3}>}$ and 0 < Θ < 1.

#### Analysis of the density of delayed airports with different *k* values

When $0<\lambda \leq \frac{N<k{>}^{2}}{W<k^{3}>}$, *i*_*k*_ ≥ 0 (*k* = 1,2,…,*n*) is the solution to Formula (15). Both sides of Formula (15) are multiplied by *kP (k)*, and the sum of 1 ≤ *k* ≤ *n* is calculated. Then, Formula (16) is obtained:

$$-i_{k}+\lambda k\frac{wk}{N<k>}(1-i_{k})\frac{1}{\langle k\rangle }\sum_{k}kP(k)\frac{Wk^{2}\lambda \Theta }{Wk^{2}\lambda \Theta +N<k>}\text{=}0$$ (15)

$$-(1-\frac{\lambda W<k^{3}>}{N<k{>}^{2}})\sum_{k}kP(k)i_{k}-\sum_{k}kP(k)i_{k}\sum_{k}kP(k)\frac{Wk^{2}\lambda \Theta }{N<k>}i_{k}\text{=}0$$ (16)

Because $1-\frac{\lambda W<k^{3}>}{N<k{>}^{2}}\geq 0$ and $\sum_{k}kP(k)i_{k}\sum_{k}kP(k)\frac{Wk^{2}\lambda \Theta }{N<k>}i_{k}\text{=}0$, according to Formula (16), *i*_*k*_ = 0(*k* = 1,2,…,*n*).

When $\lambda \geq \frac{N<k{>}^{2}}{W<k^{3}>}$is applied, the solution of Formula (13) satisfies 0 < Θ < 1. Let $i_{k}=\frac{Wk^{2}\lambda \Theta }{Wk^{2}\lambda \Theta +N<k>}$, and then 0 < *i*_*k*_ < 1. Substituting Formula (13) into Formula (15), we have

$$\text{-}\frac{Wk^{2}\lambda \Theta }{Wk^{2}\lambda \Theta +N<k>}(\Theta -<k{>}^{-1}\sum_{k}kP(k)\frac{Wk^{2}\lambda \Theta }{Wk^{2}\lambda \Theta +N<k>})=0$$ (17)

In other words, *i*_*k*_ ≥ 0 (*k* = 1,2,…,*n*) is the non-trivial solution of Formula (15).

In addition, if *i*_*k*_ ≥ 0 (*k* = 1,2,…,*n*) is the non-trivial solution of Formula (15), $\Theta =\sum_{k}kP(k)<k{>}^{-1}\frac{Wk^{2}\lambda \Theta }{Wk^{2}\lambda \Theta +N<k>}$, $i_{k}=\frac{Wk^{2}\lambda \Theta }{Wk^{2}\lambda \Theta +N<k>}$, and Θ are the only non-trivial solutions of Formula (13). Then Formula (15) has the only non-trivial solution, and 0 < *i*_*k*_ < 1 (*k* = 1,2,…,*n*).

To conclude, the equilibrium point of the delayed airport density in an airport network is $i_{k}^{s}=0$(*k* = 1,2,…,*n*) if $0<\lambda \leq \frac{N<k{>}^{2}}{W<k^{3}>}$. There is a unique non-trivial equilibrium point in the SIS model ($i_{k}^{s}\geq 0$, *k* = 1,2,…,*n*) if $\lambda \geq \frac{N<k{>}^{2}}{W<k^{3}>}$. From $\Theta =\sum_{k}kP(k)<k{>}^{-1}\frac{Wk^{2}\lambda \Theta }{Wk^{2}\lambda \Theta +N<k>}$ and $i_{k}=\frac{Wk^{2}\lambda \Theta }{Wk^{2}\lambda \Theta +N<k>}$, it can be deduced that $i_{\text{1}}^{s}<i_{2}^{s}<\cdots <i_{n}^{s}$. This indicates that when delay propagation reaches a steady state in an airport network, the delay ratio of airports with high degree values is larger than that of airports with low degree values. In addition, as the degree value increases, the delay ratio approaches 1. In other words, almost all hub airports with high degree values in the network are delayed after the flight delay becomes stable.

#### Analysis of the delayed airport density at time *t*

If the initial time is *t*_*0*_, then *i*_*k*_*(t)* (*k* = 1,2,…,*n*) denotes the delay ratio of the airports with different *k* values at *t*. The initial ratio of the delayed airports satisfies 0 ≤ *i*_*k*_ *(t*_0_*)* ≤ 1 (*k* = 1,2,…,*n*).

Formula (18) can be derived from Formula (4):

$$i_{k}(t){\text{=i}}_{k}(t_{0})\text{+}\int_{t_{0}}^{t}\left\{-i_{k}(s)+\lambda \frac{Wk^{2}}{N<k>}(1-i_{k}(s))<k{>}^{-1}\sum_{k}kP(k)i_{k}(s)\right\}ds$$ (18)

Not all *i*_*k*_*(t*_0_*)* values are 0 according to assumptions, so we have:

$$\sum_{k}kP(k)i_{k}(t_{\text{0}})>0$$ (19)

If *i*_*k*_*(t*_0_*)* = 0, then the following formula can be obtained from Formula (19):

$$-i_{k}(t_{0})+\lambda \frac{Wk^{2}}{N<k>}(1-i_{k}(t_{0}))<k{>}^{-1}\sum_{k}kP(k)i_{k}(t_{0})>0$$ (20)

Thus, there is $t'>t_{0}$ making Formula (21) valid when $t_{0}\leq t\leq t'$.


$$-i_{k}(t)+\lambda \frac{Wk^{2}}{N<k>}(1-i_{k}(t))<k{>}^{-1}\sum_{k}kP(k)i_{k}(t)>0$$ (21)

It can be seen from Formulas (18) and (21) that there is $t_{\text{0}}<t_{k}\leq t'$ that makes *0* < *i*_*k*_ *(t)* < 1 when *t*_*0*_ < *t* ≤ *t*_*k*_.

If *i*_*k*_(*t*_0_) = 1. Thus, $t'>t_{0}$ makes Formula (22) valid when $t_{\text{0}}\leq t\leq t'$.


$$-i_{k}(t)+\lambda \frac{Wk^{2}}{N<k>}(1-i_{k}(t))<k{>}^{-1}\sum_{k}kP(k)i_{k}(t)<0$$ (22)

According to Formulas (18) and (22), there is $t_{\text{0}}<t_{k}\leq t'$ that makes 0 < *i*_*k*_ *(t)* < 1 when *t*_*0*_ < *t* ≤ *t*_*k*_.

If *0* < *i*_*k*_ (*t*_0_) < 1, there is *t*_*0*_ < *t*_*k*_ that makes *0* < *i*_*k*_ (*t*) < 1 when *t*_0_ < *t* ≤ *t*_*k*_ based on the continuity of *i*_*k*_*(t)*.

In summary, if not all ratios of initially delayed airports *i*_*k*_(*t*_0_) are 0, *0* < *i*_*k*_ *(t)* < 1 for all *k* when *t* > *t*_0_. This demonstrates that in an airport network, regardless of the number airports that are initially delayed, airports with any degree value will be influenced by flight delay propagation at any time as long as there is a delayed airport at the initial time.

#### Analysis of the range of *i*_*k*_*(t)* at time *t*

Based on *0* < *i*_*k*_ (*t*) < 1, the range of delayed airport density *i*_*k*_*(t)* was further studied. To simplify the description, four theorems are proposed in this study to explore the range of *i*_*k*_*(t)* at time *t*, and their validity is proved.

**Theorem 1**: If $\underset{t\to \infty }{\lim}\sup\;i_{k}(t)\leq s_{k}$ (*s*_*k*_ ≥ 0) for each *k*, then Formula (23) is rational.


$$\underset{t\to \infty }{\lim}\sup\;i_{k}(t)\leq \frac{\frac{\lambda k^{\text{2}}W}{N<k{>}^{2}}\sum_{k}kP(k)s_{k}}{1+\frac{\lambda k^{\text{2}}W}{N<k{>}^{2}}\sum_{k}kP(k)s_{k}}$$ (23)

Theorem 1 can be proved as follows.

*i*_*k*_ *(t)* ≤ *s*_*k*_ + *ε* when *t* ≥ *τ* since $\underset{t\to \infty }{\lim}\sup\;i_{k}(t)\leq s_{k}$, ∀*ε* > 0, and ∃*τ* > *t*_0_. It has been shown above that *0* < *i*_*k*_ *(t)* < 1 applies to all *k* values. According to (4), we have

$$\frac{di_{k}(t)}{dt}\leq -i_{k}(t)+\lambda \frac{Wk^{2}}{N<k{>}^{2}}(1-i_{k}(t))\sum_{k}kP(k)(s_{i}+\varepsilon )$$ (24)

After reorganizing the above equation, Formula (25) is acquired.


$$\frac{di_{k}(t)}{dt}\leq -(1+\lambda \frac{Wk^{2}}{N<k{>}^{2}}\sum_{k}kP(k)(s_{i}+\varepsilon ))i_{k}(t)+\lambda \frac{Wk^{2}}{N<k{>}^{2}}\sum_{k}kP(k)(s_{i}+\varepsilon )$$ (25)

By multiplying both sides of Formula (25) by $\exp\{(1+\lambda \frac{Wk^{2}}{N<k{>}^{2}}\sum_{k}kP(k)(s_{i}+\varepsilon ))t\}$ and calculating the integration, Formula (26) is obtained.


$$i_{k}(t)\leq i_{k}(t_{0})\exp\{\left(1+\lambda \frac{Wk^{2}}{N<k{>}^{2}}\sum_{k}kP(k)(s_{i}+\varepsilon ))(t-t_{0}\right)\}\text{+}\frac{\frac{\lambda k^{\text{2}}W}{N<k{>}^{2}}\sum_{k}kP(k)(s_{k}+\varepsilon )}{1+\frac{\lambda k^{\text{2}}W}{N<k{>}^{2}}\sum_{k}kP(k)(s_{k}+\varepsilon )}\times (1-\exp\{\left(1+\lambda \frac{Wk^{2}}{N<k{>}^{2}}\sum_{k}kP(k)(s_{i}+\varepsilon ))(t-t_{0}\right)\})$$ (26)

Taking the upper limits of both sides of Formula (26), we have:

$$\underset{t\to \infty }{\lim}\sup\;i_{k}(t)\leq \frac{\frac{\lambda k^{\text{2}}W}{N<k{>}^{2}}\sum_{k}kP(k)(s_{k}+\varepsilon )}{1+\frac{\lambda k^{\text{2}}W}{N<k{>}^{2}}\sum_{k}kP(k)(s_{k}+\varepsilon )}$$ (27)

Let *ε* → 0, and Formula (23) is proved to be tenable.

The rationality of Theorem 2 can be verified using the same process, proving the validity of Theorem 1.

Theorem 2: If $\underset{t\to \infty }{\lim}\inf\;i_{k}(t)\geq s_{k}$ (*s*_*k*_ ≥ 0) for each *k*, then Formula (28) is workable.


$$\underset{t\to \infty }{\lim}\inf\;i_{k}(t)\geq \frac{\frac{\lambda k^{\text{2}}W}{N<k{>}^{2}}\sum_{k}kP(k)s_{k}}{1+\frac{\lambda k^{\text{2}}W}{N<k{>}^{2}}\sum_{k}kP(k)s_{k}}$$ (28)

#### Global stability analysis

The SIS model for flight delay propagation in an airport network is shown in Formula (4). Based on the analysis above, when $0<\lambda \leq \frac{N<k{>}^{2}}{W<k^{3}>}$, Formula (4) has only one equilibrium point, namely, the zero solution. When $\lambda >\frac{N<k{>}^{2}}{W<k^{3}>}$, Formula (4) has two equilibrium points. One is a zero solution and the other is the unique non-trivial solution $i_{k}^{s}>0$ (*k =* 1,2,*…*,*n*) of Formula (29).


$$-i_{k}(t)+\lambda \frac{Wk^{2}}{N<k>}(1-i_{k}(t))<k{>}^{-1}\sum_{k}kP(k)i_{k}(t)\text{=}0$$ (29)

Let ${\overset{―}{i}}_{k}^{\left(1\right)}=1$ (1 ≪ *k* ≪ *n*), the following sequence is defined:

$${\overset{―}{i}}_{k}^{\left(m+1\right)}=\frac{\frac{\lambda k^{\text{2}}W}{N<k{>}^{2}}\sum_{k}kP(k){\overset{―}{i}}_{k}^{\left(m\right)}}{1+\frac{\lambda k^{\text{2}}W}{N<k{>}^{2}}\sum_{k}kP(k){\overset{―}{i}}_{k}^{\left(m\right)}},1\ll k\ll n,m=1,2,\ldots$$ (30)

Because of $\underset{t\to \infty }{\lim}\inf\;i_{k}(t)\leq 1\text{=}{\overset{―}{i}}_{k}^{\left(1\right)}$(1 ≪ *k* ≪ *n*), Formula (31) can be derived from Formula (30) based on Theorem 1.


$$\underset{t\to \infty }{\lim}\sup\;i_{k}(t)\leq {\overset{―}{i}}_{k}^{\left(m\right)},1\ll k\ll n,m=1,2,\ldots$$ (31)

If not all *i*_*k*_*(t*_0_*)* values are 0, $\underset{t\to \infty }{\lim}\inf\;i_{k}(t)>0$ for each *k*. Therefore, $0<{\underset{―}{i}}_{k}^{\left(1\right)}<\underset{t\to \infty }{\lim}\inf\;i_{k}(t)$ is performed, and the following sequence is defined:

$${\underset{―}{i}}_{k}^{\left(m+1\right)}=\frac{\frac{\lambda k^{\text{2}}W}{N<k{>}^{2}}\sum_{k}kP(k){\underset{―}{i}}_{k}^{\left(m\right)}}{1+\frac{\lambda k^{\text{2}}W}{N<k{>}^{2}}\sum_{k}kP(k){\underset{―}{i}}_{k}^{\left(m\right)}},1\ll k\ll n,m=1,2,\ldots$$ (32)

Based on Theorem 2, Formula (32) can be derived from Formula (31).


$$\underset{t\to \infty }{\lim}\inf\;i_{k}(t)\geq {\underset{―}{i}}_{k}^{\left(m\right)},1\ll k\ll n,m=1,2,\ldots$$ (33)

According to Formulas (31) and (33), we have the following:

$${\underset{―}{i}}_{k}^{\left(m\right)}\leq \underset{t\to \infty }{\lim}\inf\;i_{k}(t)\leq \underset{t\to \infty }{\lim}\sup\;i_{k}(t)\leq {\overset{―}{i}}_{k}^{\left(m\right)},1\ll k\ll n,m=1,2,\ldots$$ (34)

Because ${\underset{―}{i}}_{k}^{\left(m\right)}$ monotonically increases and converges to $i_{k}^{s}$, and ${\overset{―}{i}}_{k}^{\left(m\right)}$ monotonically decreases and converges to $i_{k}^{s}$, let *m* → ∞ in Formula (34), and $i_{k}^{s}\leq \underset{t\to \infty }{\lim}\inf\;i_{k}(t)\leq \underset{t\to \infty }{\lim}\sup\;i_{k}(t)\leq i_{k}^{s}$ is obtained. That is, $\underset{t\to \infty }{lim}\;i_{k}(t)=i_{k}^{s}.$

The above results indicate that when $\lambda >\frac{N<k{>}^{2}}{W<k^{3}>}$, flight delay propagation enters the steady state, and the ratios of delayed airports with different degree values in an airport network converge to the equilibrium point. If not, all the ratios of initially delayed airports in the network are 0.

### Analysis of the delay propagation scale

During delay propagation in an airport network, the delay propagation rate *λ* and degree distribution of the network *P(k)* are two key parameters that determine the equilibrium point of the delay propagation in the steady state. The propagation rate *λ* reflects the delay property with flights as the medium. The degree distribution of the network *P(k)* describes the topological structure of the network. Therefore, when the system tends to be stable, the ratio of delayed airports with different degree values *i*_*k*_ in an airport network is determined by the delay propagation intensity and the topological structure of the network.

To further investigate the influence of the delay propagation intensity and topological structure of an airport network on delay propagation, Theorem 3 is proved to be true as follows.

**Theorem 3**: Supposing $\lambda >\frac{N<k{>}^{2}}{W<k^{3}>}$, $g(x,\lambda ,P)=\sum_{k}kP(k)<k{>}^{-1}\frac{\frac{\lambda Wk^{2}x}{N<k>}}{1+\frac{\lambda Wk^{2}x}{N<k>}}-x$ has the following properties.

There is a unique *x*_*p*_ > 0 that makes *g(x*_*p*_,*λ*,*P)* = 0. *x*_*p*_ > 0, $g_{x}^{\prime }\left(x_{p},\lambda ,P\right)<0$.

If there is *γ* > 0 that makes *P* (*k*)∝*k*^*-γ*^, then $g_{\gamma }^{'}(x_{p},\lambda ,P)<0$ for random *λ* > 0 and *x* > 0.

The validity of Theorem 3 can be proven as follows.

Let *f (x)* = *g(x*, *λ*, *P)* for a fixed $\lambda >\frac{N<k{>}^{2}}{W<k^{3}>}$ and degree distribution *P*, and the derivative of *f (x)* is calculated as

$$f^{\prime }(x)=\underset{k}{\sum }kP(k)<k{>}^{-1}\frac{\frac{\lambda Wk^{2}}{N<k>}}{{\left(1+\frac{\lambda Wk^{2}x}{N<k>}\right)}^{2}}-1,$$

$$f^{\prime \prime }(x)=-2\underset{k}{\sum }kP(k)<k{>}^{-1}\frac{\frac{\lambda^{2}W^{2}k^{3}}{N^{2}<k{>}^{2}}}{{\left(1+\frac{\lambda Wk^{2}x}{N<k>}\right)}^{3}}$$ (35)

According to Formula (35), when *x* ≥ 0,

$$f^{\prime }(0)=\lambda \frac{W<k^{3}>}{N<k{>}^{2}}-1>0,$$

$$f^{\prime \prime }(0)=-2\underset{k}{\sum }kP(k)<k{>}^{-1}\frac{\lambda^{2}W^{2}k^{3}}{N^{2}<k{>}^{2}}<0$$ (36)

Bringing *P* (*k*)∝*k*^*-γ*^ to *g(x*,*λ*,*P)*, we have:

$$g(x,\lambda ,P)\text{=}\frac{\sum_{k=1}^{N}\frac{\lambda Wk^{3\text{-}\gamma }x}{N\sum_{k=1}^{N}k^{1-\gamma }+\lambda Wk^{2}x}}{\sum_{k=1}^{N}k^{1-\gamma }}-x$$ (37)

It can be calculated from Formula (37) that $g_{\gamma }^{'}(x,\lambda ,P)<0$, which proves that the second property of Theorem 3 is tenable.

To further discuss the influence of the delay propagation intensity and topological structure of an airport network on the ratio of delayed airports with different degrees of *i*_*k*_ in a network in the steady state, the relation of *i*_*k*_ with *λ* and *P(k)* is represented by the function *i*_*k*_*(λ*,*P)*. Formula (38) is derived from Formula (6).


$$i_{k}(\lambda ,P)=\frac{1}{\langle k\rangle }\sum_{k}kP(k)\frac{Wk^{2}\lambda i_{k}(\lambda ,P)}{Wk^{2}\lambda i_{k}(\lambda ,P)+N<k>}$$ (38)

According to Formula (37), *i*_*k*_*(λ*,*P)* > 0, we have

$$g(i_{k}(\lambda ,P),\lambda ,P)=0$$ (39)

Due to the scale-free feature of the topological structure of an airport network, the degree distribution conforms to the power-law distribution pattern of *P (k)* ∝ *k*^*-γ*^. Supposing there is *γ* > 0, the partial derivatives of *γ* on both sides of Formula (39) are solved, and Formula (40) is obtained.


$$g_{x}^{'}(i_{k}(\lambda ,P),\lambda ,P)i_{k}{}_{\gamma }^{'}(\lambda ,P)+g_{\gamma }^{'}(i_{k}(\lambda ,P),\lambda ,P)=0$$ (40)

Based on Theorem 3, $i_{k}{}_{\gamma }^{'}(\lambda ,P)<0$ can be obtained using Formula (40). According to Formula (38), with an increasing delay propagation rate, the ratio of delayed nodes to total nodes with different degree values increases, and the probability of linking to delayed airports through a random route also increases. The ratio of delayed airports with different degrees is a decreasing function of the degree distribution index *γ*. A smaller degree distribution index indicates a larger delay propagation rate, a greater difference in the degree values of airports, and stronger scale-free attribute of the network. This indicates that the scale-free property promotes delay propagation.

## Discussion

**Fig 1:**
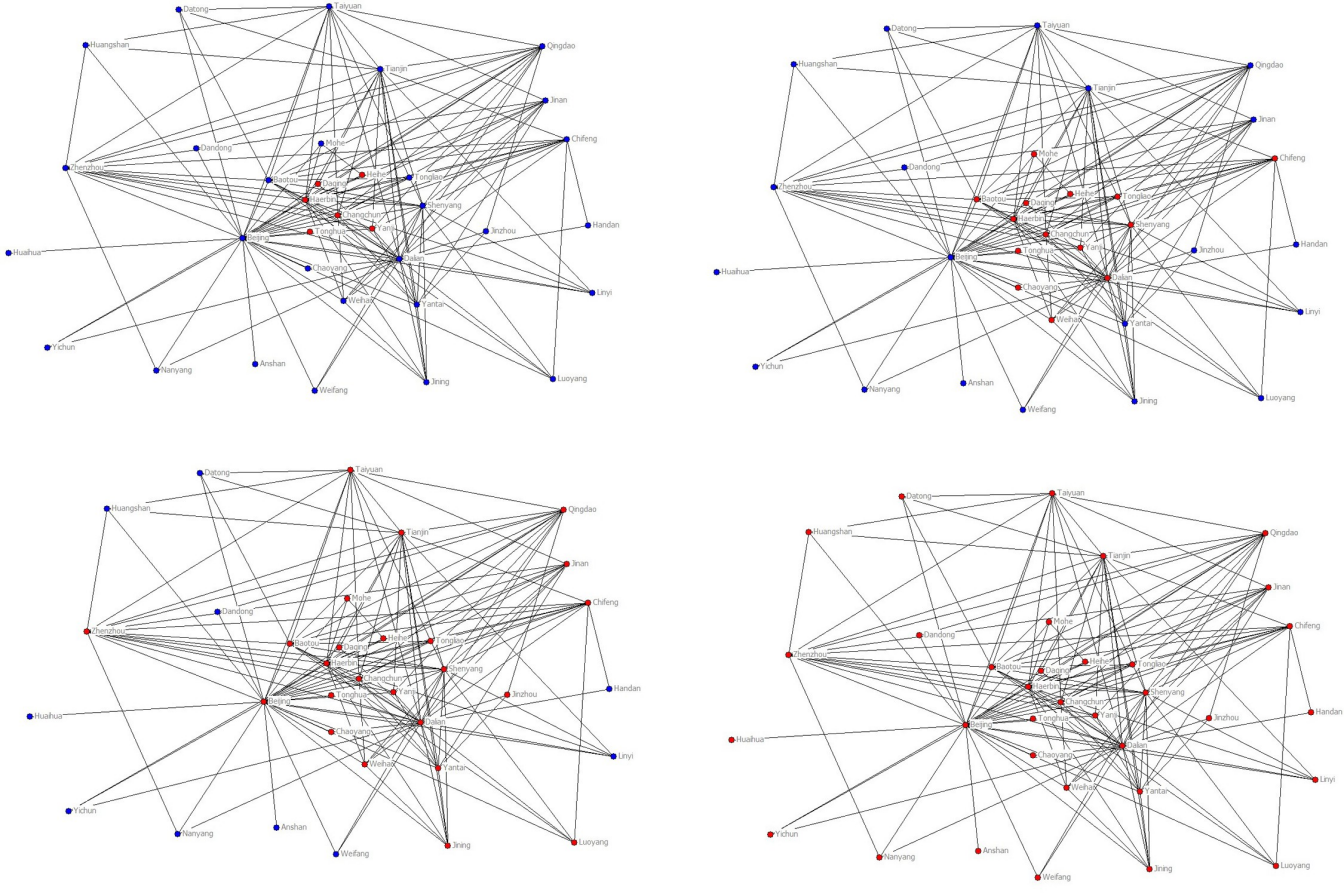
The propagation of flight delays in China in 2017. (**a**) Airports affected by heavy snow, (**b**) Airports affected by the delay propagation on the first day, (**c**) Airports affected by the delay propagation on the second day, (**d**) Airports affected by the delay propagation on the third day.

On February 7, 2017, heavy snowfall in Northeast China caused the closure of multiple airports in Heilongjiang and Jilin provinces. Due to the peak period of air passenger travel at that time, there was a spread of flight delays. In the next three days, 34 Chinese airports of seven provinces have been affected by the spread of this delay, resulting in flight delays, as shown in Fig 1. In the figure, the delayed airport is marked in red.

We illustrate the severity of delay propagation in airport networks through an example from China. In the previous study, by grouping airports according to degree values, we constructed a flight delay density equation in the airport network based on a heterogeneous SIS model, and determined the critical conditions for delay propagation in the airport network. The focus was on three main issues: "the range of values for delayed airport density", "the convergence interval of delayed airport density with different degrees", and "the scale of airports with flight delays in the network". These three issues are important characteristics of delay propagation in the airport network after reaching a stable state. In addition, due to the limitations of analytical methods, it is necessary to further reveal the steady-state characteristics of delay propagation in the airport network through simulation methods.

Below, we will generates a simulated airport network with scale-free attributes, design a simulation system, and verify the critical value of delay propagation. Through simulation methods, further discuss the impact of "initial delay airport number", "λ", and "network structure" on delay propagation after reaching a steady state.

### Generation of the scale-free virtual airport network

The degree distribution of nodes in a scale-free network aligns with the power-law distribution pattern. The BA model proposed by Barabási and Albert was used as the basic model to generate scale-free networks. The BA scale-free network model was constructed based on network growth and preferential connections using the following algorithm:

① Network growth: Each time a new node is introduced into the network with *m*_*0*_ nodes, that node is linked to *m* existing nodes, *m* ≤ *m*_*0*_.
② Preferential connection: *j* is a new node, and *i* is an existing node in the network. The relationship between the probability *Π*_*i*_, where *j* is connected to *i*, and the degree values (*k*_*i*_ and *k*_*j*_) of *j* and *i* is expressed as

$$\Pi_{i}=\frac{k_{i}}{\sum_{j}k_{j}}$$ (41)

After *t* iterations, the algorithm produced a network with *N* = *t*+*m*_0_ nodes and *mt* edges.

**Fig 2:**
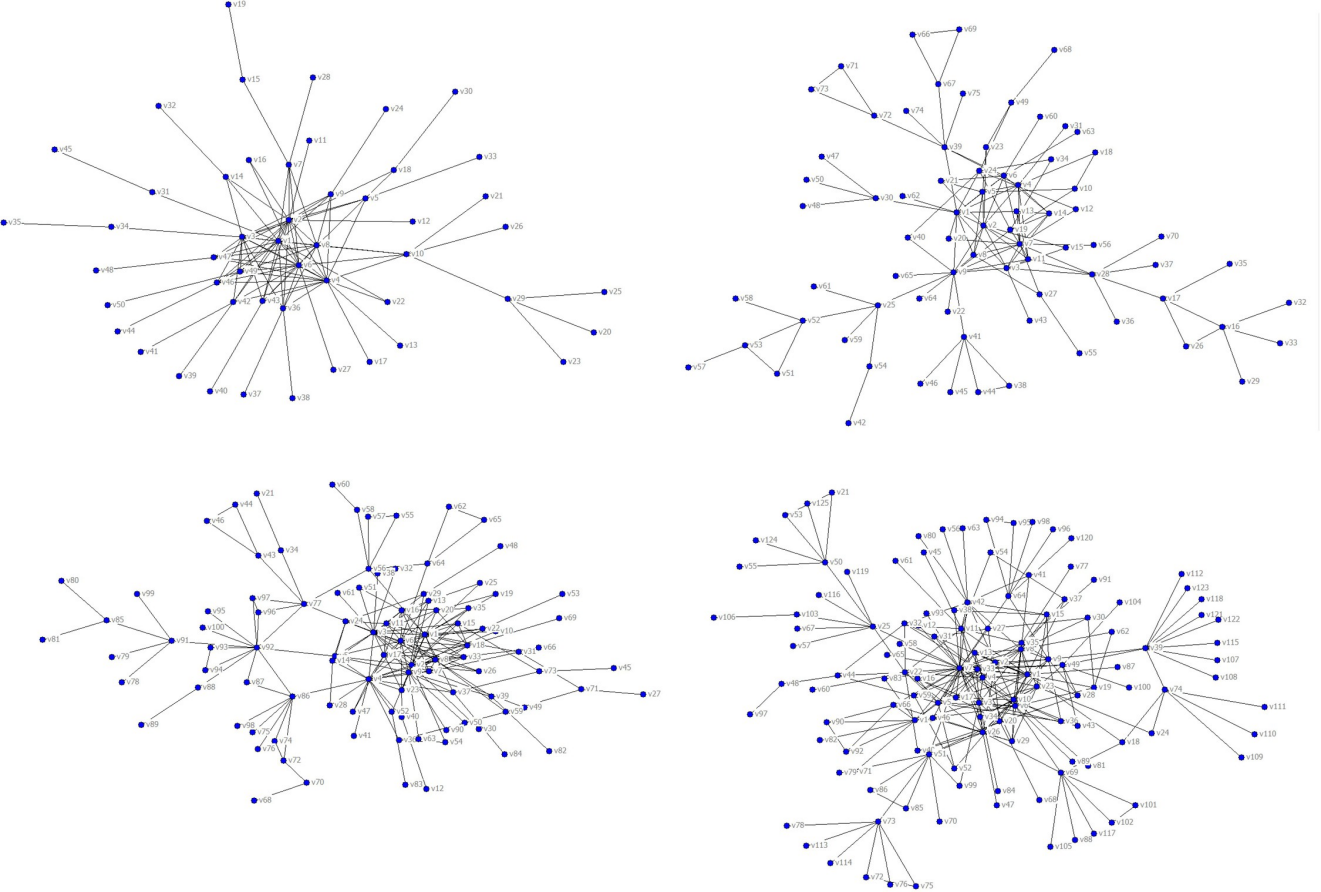
Virtual airport networks. (**a**) Virtual airport network, A_1_, (**b**) Virtual airport network, A_2_, (**c**) Virtual airport network, A_3_, (**d**) Virtual airport network, A_4_.

To reveal delay propagation features in different scale-free networks, PAJEK software was used to generate four virtual airport networks of different sizes, namely A_1_, A_2_, A_3_, and A_4_, according to the generation algorithm of the above BA scale-free network model and the parameters in Table 1, as shown in Fig 2.

**Table 1:** Parameters of the BA scale-free network model.

Parameter	Value
Maximum number of nodes	50, 75, 100, 125
Maximum number of edges	150, 225, 400, 500
Node average degree	3.00, 3.00, 4.00, 4.00
Number of nodes in the initial graph	10
Node connection probability in the initial graph	0.60
Alpha value	0.30
Beta value	0.30

The number of airports, number of routes, average degree, degree distribution index, and other basic parameters of the four virtual airport networks were calculated using UCINET software, as shown in Table 2.

**Table 2:** Basic parameters of virtual airport networks.

	Airport No.	Route No.	<*k*>	<*k*^3^>	*γ*
A_1_	50	105	4.2	581.76	3.10
A_2_	75	124	3.31	230.15	2.13
A_3_	100	195	3.98	526.41	2.01
A_4_	125	254	4.06	733.38	2.36

To roughly balance the flight density and e ratio of initially delayed airports across the A_1_, A_2_, A_3_, and A_4_ virtual airport networks, the initial parameters of their delay propagation simulation systems are determined as shown in Table 3.

**Table 3:** Initial parameters of flight delay diffusion propagation systems.

Parameter	Value
Scale-free network adjacency matrix	0–1 matrixes corresponding to A_1_, A_2_, A_3_, and A_4_ networks
Network route distance matrix	Each matrix element is multiplied by a random number of [0, 100]
Flight flow direction and quantity	If there are five two-way flights for each route, the total number of flights in A_1_, A_2_, A_3_ and A_4_ networks is 1050, 1240, 1950 and 2540, respectively
Flight flow rate	Each matrix element is multiplied by a random number of [0, 50]
Time scale	1
Number of initially delayed airports	17, 25, 33, 41

### Simulation test of delay propagation features

#### Simulation test of the delay propagation critical condition

**Fig 3:**
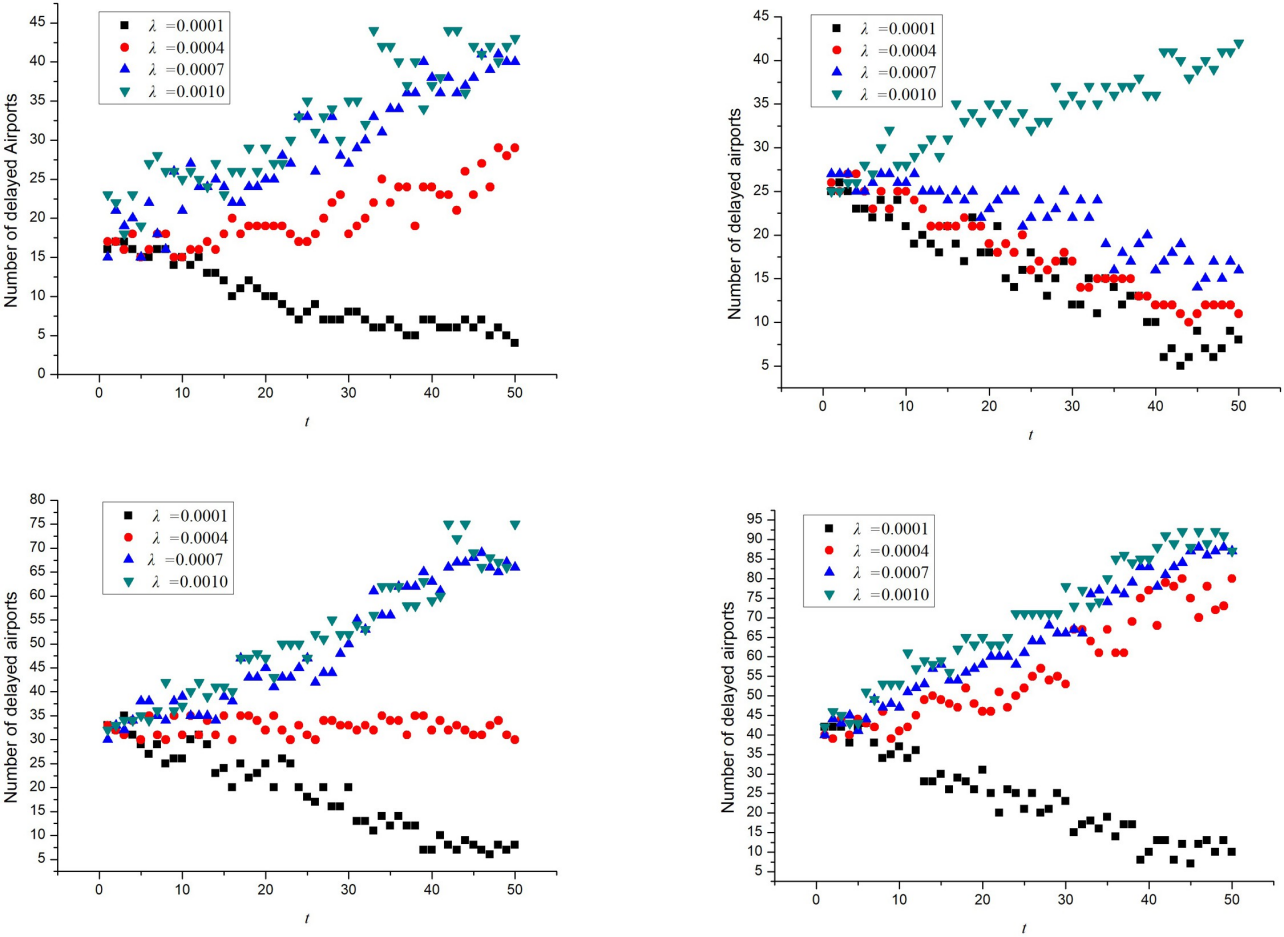
Variation in the number of delayed airports. (**a**) A_1_, (**b**) A_2_, (**c**) A_3_, (**d**) A_4_.

In the simulation system, the initially delayed airport and the departure times of the original and newly generated flights were generated randomly and independently from each other. During the simulation, *λ* was set to 0.0001, 0.0004, 0.0007, and 0.0010 for networks A_1_, A_2_, A_3_, and A_4_, respectively. To eliminate the influence of accidental factors, the average value of five simulation results was obtained. The changes in the number of delayed airports in networks A_1_, A_2_, A_3_, and A_4_ networks at 50 different moments are shown in Fig 3.

From Formula (12), Tables 2 and 3, the delay propagation critical values of the four networks were calculated to be *λ*_*c*1_ = 0.00034, *λ*_c2_ = 0.00087, *λ*_c3_ = 0.00039, and *λ*_c4_ = 0.00027. In Fig 3, when *λ > λ*_*c*_, delay propagation occurs in the airports of an airport network. The simulation results are in good agreement with Formula (12).

#### Simulation test of delay propagation in steady state

**Fig 4:**
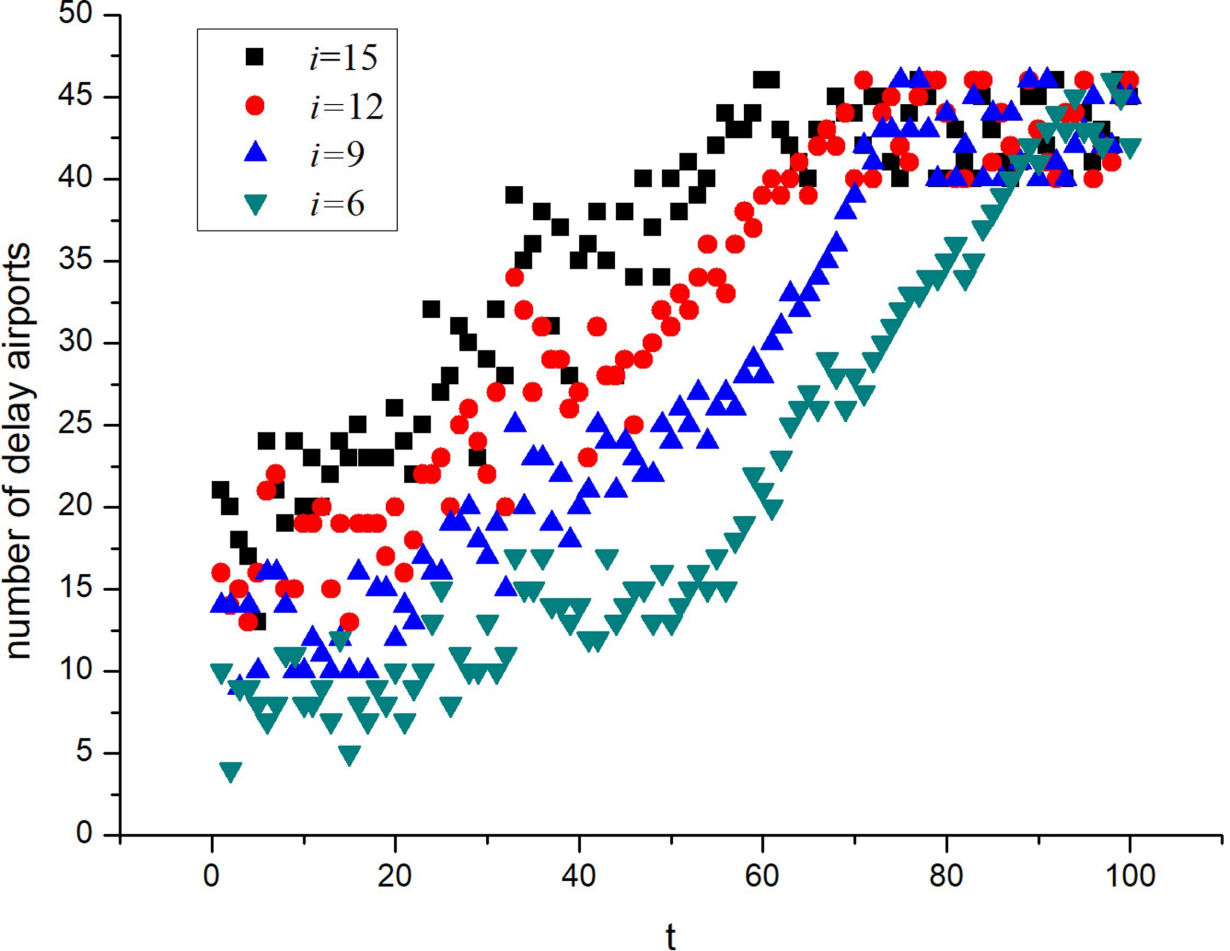
Delay propagation in the A_1_ network for different numbers of initially delayed airports.

To facilitate this research, simulation experiments were carried out on the A_1_ network, which had the fewest airports. *λ* was set to 0.0010, which exceeds the critical condition of the delay propagation. The number of initially delayed airports was set to 6, 9, 12, and 15, and the other parameter settings are listed in Table 3. The average of the five simulation results was calculated when the number of initially delayed airports was different, as shown in Fig 4.

It can be observed from Fig 4 that the delay propagates as long as the delay propagation critical value is exceeded, regardless of the number of airports that are initially delayed. Moreover, when the delay propagation reaches a steady state, the ratio of delayed airports in the network remains basically the same. Different numbers of initially delayed airports affect the time required to reach a steady state. A small number of initially delayed airports means a longer time to reach a steady state.

**Fig 5:**
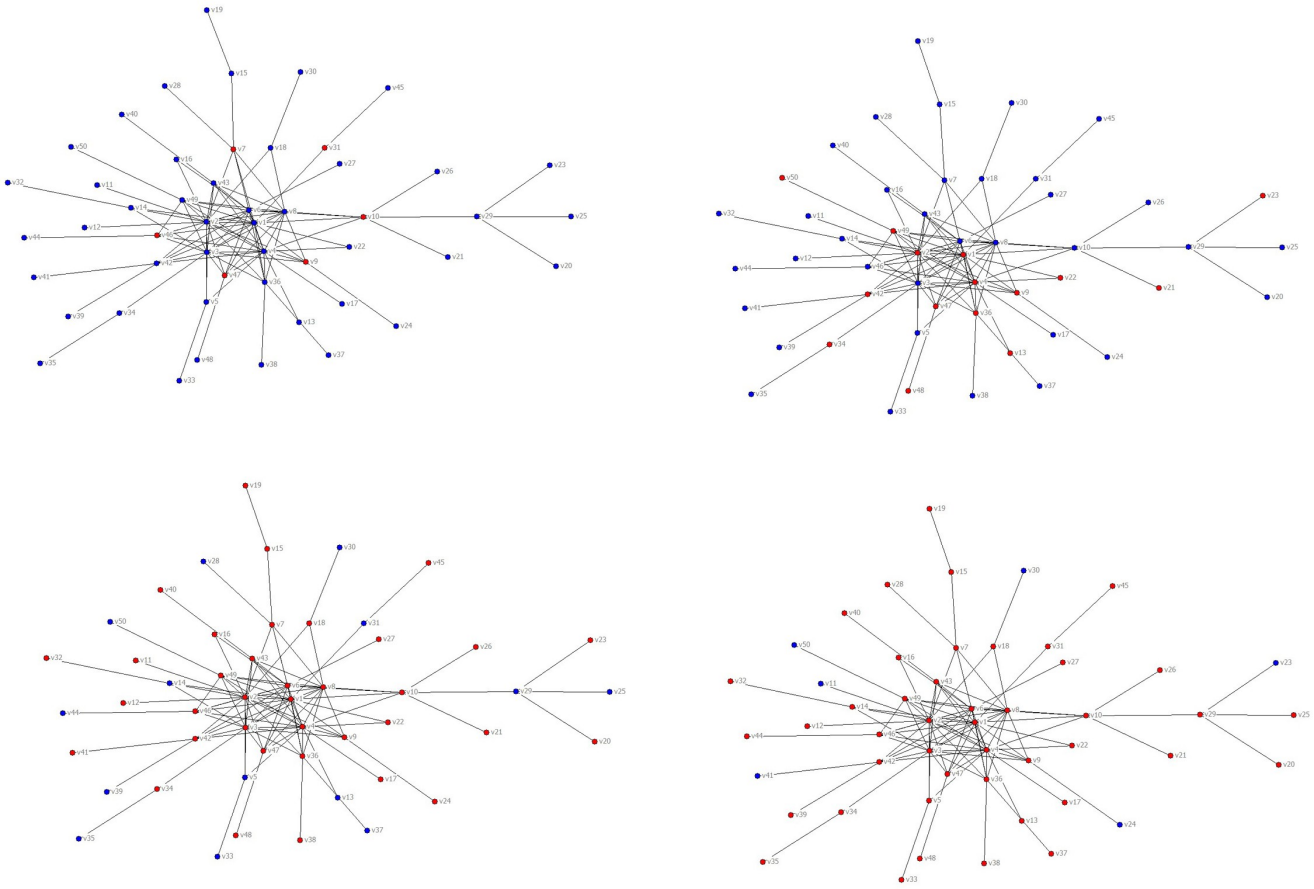
Delay diffusion in A_1_ at different times. (**a**) t = 0, (**b**) t = 40, (**c**) t = 80, (**d**) t = 100.

The number of initially delayed airports was set to six in the A_1_ network. The delay propagation of airports at different times is shown in Fig 5. In the figure, normal nodes are marked in blue and delayed nodes are marked in red.

In Fig 5, nodes with higher degree values are notably more likely to be delayed compared to those with lower degree values. Nodes with higher degree values are difficult to restore to normal once they are delayed. Additionally, even when the number of initially delayed airports is the same, different initially delayed airports result in different delay propagation rates.

#### Simulation influence of *λ* on the delay propagation scale

An increased delay propagation rate *λ* increases the probability that the delayed airport affects other connected airports. According to Formula (38), as the delay propagation rate increases, the ratio of delayed nodes to all nodes with different degree values in the network increases, and the scale of delayed nodes in the steady state also increases.

**Fig 6:**
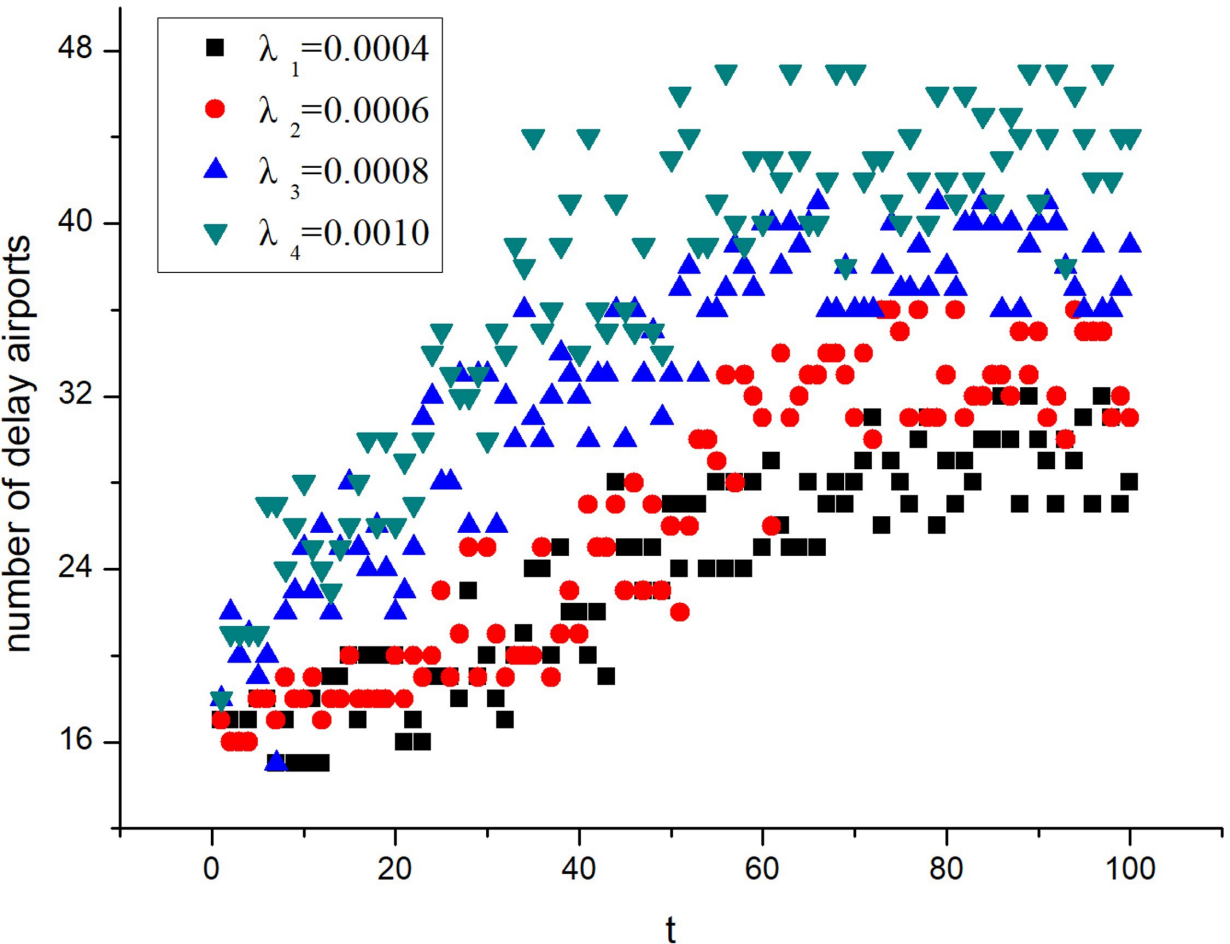
Influence of *λ* of A_1_ network on the delay propagation scale.

To simplify the research, simulation tests were conducted on the A_1_ network, and the number of initially delayed airports was set to 17. The delayed-propagation critical value of the A_1_ network is *λ*_*c*1_ = 0.00034; therefore *λ*_1_ = 0.0004, *λ*_2_ = 0.0006, *λ*_3_ = 0.0008, and *λ*_4_ = 0.0010. The average values of the five simulation results were calculated. The node delay propagation in the A_1_ network at different ***λ*** values is shown in Fig 6.

It is evident from Fig 6 that with an increase in ***λ***, the time taken by the delayed nodes to reach the steady state is shortened constantly in the network, and the scale of the delayed nodes in the steady state increases continuously. The simulation results agree with the conclusion of Formula (38).

#### Influence of the network structure on the delay propagation scale

Flight delay propagates through routes between airports, and thus the airport network structure directly affects delay propagation. The degree distribution index *γ* is a vital indicator of network structure. The smaller the value of *γ*, the greater the difference in the degree values of the nodes, and the stronger the scale-free attribute of the network. The value of *γ* can be determined from the node degree distribution in a network. The values of *γ* are 3.10, 2.13, 2.01, and 2.36, respectively, in the A_1_, A_2_, A_3_, and A_4_ networks. The difference in node degree values was the greatest in the A_3_ network and the smallest in the A_1_ network.

**Fig 7:**
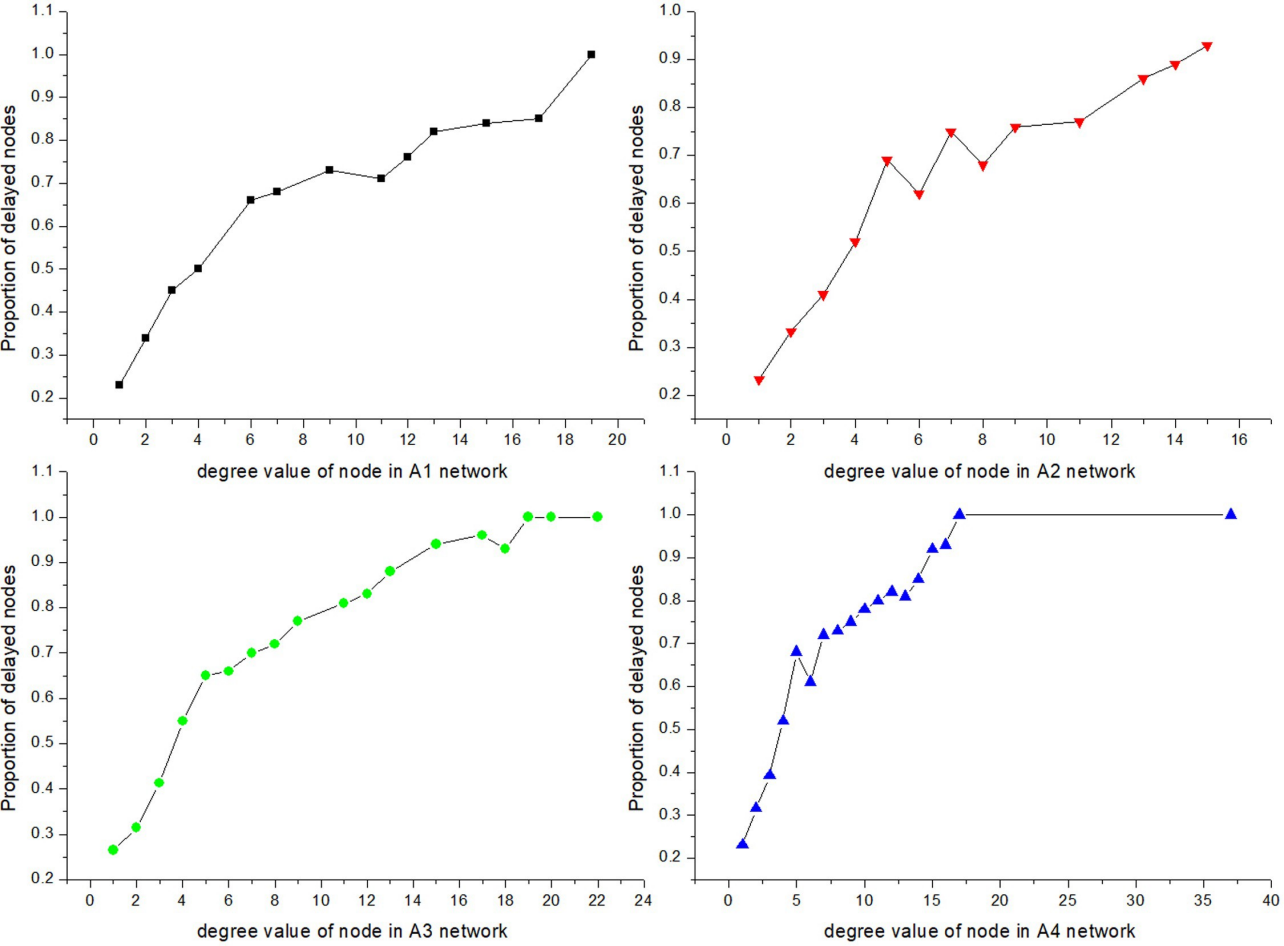
Density of delayed airports with different degree values.

Taking *λ =* 0.0010, the system simulation was carried out by setting the number of initially delayed airports as 17, 25, 33, and 41 in the A_1_, A_2_, A_3_, and A_4_ networks, respectively. To avoid the influence of accidental factors, the average value of five times simulation results was calculated. The densities of delayed airports with different degree values in the A_1_, A_2_, A_3_, and A_4_ networks are illustrated in Fig 7.

In Fig 7, the ratio of delayed airports increases with an increase in the degree value. The delay ratio of airports with higher degree values is far higher than that of airports with lower degree values. In general, the delay ratio of nodes of the same degree value is highest in the A_3_ network and lowest in the A_1_ network. This indicates that the delay ratio of airports with various degrees in the airport network is a decreasing function of the degree distribution index *γ*. In other words, the smaller the degree distribution index, the larger the delay propagation ratio. Hence, the scale-free attribute of the network promotes delay propagation, which verifies the reliability of the results of Formula (40).

## Conclusions

The entire process from generation and propagation to the disappearance of delays in the airport network is explored in this study. A density equation of delayed airports at time *t* was established to solve the critical condition of delay propagation in airport networks. The steady-state features and scale of the delay propagation were investigated. Finally, a simulation system was designed and simulation experiments were conducted to verify the reliability of the research results. The following conclusions with regard to airport networks were drawn from this study.

1. The critical value of delay propagation is directly proportional to the number of nodes in the network and <*k*>^2^ and inversely proportional to the number of flights and <*k*^3^>. Due to the scale-free property of an airport network, *W*<*k*^3^> is far greater than *N*<*k*>^2^. Therefore, the critical value *λ*_*c*_ of delay propagation is so small that delay propagation can easily occur.
2. When the critical value is exceeded and the delay propagation reaches a steady state, the degree value of the nodes is highly related to the delay state. The delay ratio of airports with high degrees is larger than that of airports with low degrees. Moreover, as the degree value increases, the delay ratio approaches 1. When the delay propagation enters the steady state, hub airports with high degree values are the most susceptible to delays and can easily be delayed. In contrast, airports with fewer routes are less affected by delay propagation.
3. Airports of any degree value will be delayed at any time when the critical value .,m/ k of the delay propagation is exceeded as long as there is a delayed airport at the initial time, regardless of the number of initially delayed airports. In the steady state, the ratio of delayed airports in the network is essentially the same. However, the number of initially delayed airports affects the time required to reach a steady state. Fewer initially delayed airports indicate a longer time to reach a steady state.
4. If the propagation rate is smaller than the critical value of delay propagation, the final differential equation has only one equilibrium point, namely the zero solution. Under such conditions, the delay does not propagate, and with passing time, all nodes in an airport network ultimately return to the normal state. If the propagation rate is equal to or greater than the critical value of the delay propagation, the differential equation has one non-trivial solution, $i_{k}^{s}>0$. If not all ratios of initially delayed airports in an airport network are zero, the ratios of delayed airports with different degree values will converge to the equilibrium point $i_{k}^{s}$ after delay propagation reaches a steady state.
5. When the critical value of the delay propagation is exceeded, the ratio of delayed nodes to all nodes of different degree values increases, and the node delay scale also increases with an increase in the delay propagation rate *λ*. In addition, the network structure affects the delay propagation. The ratio of delayed airports of various degrees is inversely proportional to the degree distribution index *γ* in an airport network. The smaller the value of *γ*, the larger the ratio of the delay propagation. This indicates that a greater difference in the degree values between airports in the network is more conducive to delay propagation.

## Supporting information

S1 Table Adjacency matrix data of the virtual airport network. There are four sheets in this table that store the critical matrix data of four virtual airport networks: A_1_, A_2_, A_3_, and A_4_. Among them, rows and columns represent airports, 0 in the matrix represents no flight route between two airports, and 1 represents a flight route between two airports.
(XLSX)

S2 Table Summary of data in simulation. Statistics on the number of delayed airports over time in the four virtual airport networks A_1_, A_2_, A_3_, and A_4_ with different values of λ.
(XLSX)

S3 Table Summary of data in simulation. Statistics on the change of the number of delayed airports over time in the four virtual airport networks A_1_, A_2_, A_3_, and A_4_ with different initial delayed airports.
(XLSX)

S4 Table Summary of data in simulation. Influence of *λ* of A1 network on the delay propagation scale.
(XLSX)

S5 Table Summary of data in simulation. Statistics of airport delay percentages with different degree values in four virtual airport networks.
(XLSX)

S6 Table Statistical table of delayed flights. This table contains a total of 4 sheets, which respectively counted the daily delayed flight information of each airport in the northern airport network of China from February 7th to 10th, 2017.
(XLSX)
